# Development of a new preclinical model to study early implant loss: a validation study in the beagle dog

**DOI:** 10.1007/s00784-022-04642-3

**Published:** 2022-07-26

**Authors:** Antonio Liñares, Raul Verdeja, Benjamin Pippenger, Fernando Muñoz, Mónica López-Peña, Juan Blanco

**Affiliations:** 1 Periodontology Unit, School of Medicine and Dentistry, University of Santiago de Compostela, Rúa San Francisco s/n 15782, Santiago de Compostela, Spain; 2 Department of Surgery, Service of Maxillofacial and Oral Surgery, University Hospital and Faculty of Medicine, Geneva, Switzerland; 3 Preclinical & Translational Research, Institut Straumann, Basel, Switzerland; 4 Department of Veterinary Clinical Sciences, Universidade de Santiago de Compostela, Lugo, Spain

**Keywords:** Early implant loss, Animal model, Infection

## Abstract

**Objectives:**

To develop a new preclinical model to study early implant loss, where local infection conditions would impair the implant osseointegration.

**Materials and methods:**

Forty-eight smooth, 2.9-mm diameter experimental implants were placed in the mandible of 8 beagle dogs (3 in each side). In half of the animals (test group, *n* = 24 implants), the implants received ligatures around the implant-abutment connection. In the other half, no ligatures were placed (control group, *n* = 24 implants). Four weeks later, implants were extracted in a flapless approach and standard 3.3-mm diameter SLActive implants were placed into the same osteotomy site without any further drilling. Eight weeks after the second implantation, animals were sacrificed and analyzed in terms of implant survival.

**Results:**

After 8 weeks of healing, 4 implants were lost in the control group and 14 in the test group. This corresponded to a 17.4% of early implant loss in the control group and 58.3% in the test. Most of the early failures occurred within the first 5 weeks of healing.

**Conclusions:**

Implants placed in a pre-contaminated site present higher early loss than those placed in a non-contaminated site. This study represents a valid and robust preclinical model to study mechanisms and reduction of early implant loss as new technologies become available.

**Clinical relevance:**

Scientific rationale for the study: There is lack of animal models to study early implant loss. Thus, a proposal of a new model is presented. With the validation of this model, new technologies can be implemented to prevent early implant loss.

## Introduction

Over the last 20 years, dental implant treatment has continued to become more predictable and successful, with improvements in terms of survival and complications rates [1]. However, a certain number of implant failures still occur and are generally categorized into early and late implant loss. Early implant loss is defined as the biological failure of a dental implant to establish osseointegration. The term “early” specifically refers to implants that fail before prosthetic restoration (loading). Dental implant complications in dental implantology, although few in number, remain a challenging treatment. Discussions in the recent publications remain current as the etiology, treatment of péri-implantitis, and replacement are still controversially discussed [2–4]. Etiologically speaking, the principle reported reasons for early implant loss which are (1) surgical trauma, (2) microbial infection, (3) premature loading, and/or (4) impaired healing ability [5]. Three of the four factors are surgeon- or patient-related and can only be ameliorated with better surgical training or better underlying disease management (patients with diabetes, metabolic syndrome, or undergoing prolonged steroid treatment, for example). However, microbial infection is a parameter that can be directly influenced. Indeed, microbial infection is reported to be the most common reason for complications that might occur during the primary healing period (osseointegration) [6]. Considering early implant loss occurs because something interferes with the primary healing of an implant, microbial infection can therefore be considered as the primary cause of early implant loss. This means primary infections in the mouth must be controlled before any implant rehabilitation. Periodontitis without treatment may be an increase on early implant loss due to bacteria translocation. In fact, Derks [7] showed that early implant loss occurred in 1% of no periodontitis patients and 2.3% in patient having periodontitis. This is an increased odds ratio of 3.3. According to Kim et al., the survival rate of implants placed immediately after implant lost depends on the usual factors responsible for implant failure as location, removal technique, or grafting [8].

In a recent publication, Derks et al. reported on the loss of dental implants assessed in 4716 randomly selected patients [7]. In total, 596 of the subjects (representing 2367 implants) attended a clinical examination 9 years after initial implant therapy. Early implant loss, considered losses only before implant prosthesis connection, occurred in 4.4% of patients (1.4% of implants), while 4.2% of the patients presented with late implant loss (2.0% of implants). Multilevel analysis revealed higher odds ratios for early implant loss among smokers and patients with an initial diagnosis of periodontitis. Implants shorter than 10 mm and representing certain brands also showed higher odds ratios for early implant loss. Thus, this study demonstrated that early and late implant loss is similar at the patient level. Another cohort retrospective study [9] showed implant outcomes in 1727 patients with and without a history of periodontitis. Patients were screened according to the severity of the disease recorded before treatment and implant therapy. Six hundred and thirty patients were in the severe periodontitis (SP) group, 839 in the moderate periodontitis (MP) group, and 258 had no periodontitis (NP). Patients requiring periodontal treatment were treated prior to implantation. In total, 3260 implants and 1707 implant-supported prostheses were placed in the SP group, 2813 implants and 1744 implant-supported prostheses in the MP group, and 647 implants and 424 implant-supported prostheses in the NP group. At the end of the study, 250 patients were lost to the 5-year post-loading follow-up. For implant failures, 130 (4.5%) implants failed in the SP group, 74 (3.1%) implants failed in the MP group, and 15 (3.0%) implants failed in the NP group. Most of the implant failures (90%) occurred before implant loading. Using a regression analysis, the authors reported that the history of periodontitis did not affect the implant survival outcomes. Regarding immediate implant placement after dental extractions, Wagenberg et al. found in their 16 years study a 96% success rate, an early loss of 3.7%, and late after weight bearing of 0.3% [10].

While the prevalence of early implant loss reported in the literature ranges from as little as 1.2%, other reports have suggested rates as high as 4.1%. A systematic review evaluated early implant loss on short and long implants. Early implant loss was more significant in smooth than rough implants, short than long, and in the maxilla than in the mandible [8]. Clearly, although early implant loss is relatively common, little to no data exists to clarify the mechanisms. This lack of evidence stems in part from the fact that no preclinical model currently exists to study the pathogenesis and treatment options for early implant loss. Some models were designed to test if a contaminated and later cleaned implant surface can osseointegrate in the same manner as a non-contaminated one [12–14], but do not attempt to mimic the contaminated environment most likely responsible for early implant loss. Due to the emerging prevalence of peri-implantitis, most preclinical animal models involving contaminated implants have been developed to assess the pathogenesis of peri-implant lesions undergoing different disinfection therapies and/or reconstructive surgeries. However, these peri-implantitis models should be considered as too harsh of a defect to study early implant loss. Early implant loss does not necessarily imply saucerization bone defects or even soft tissue breakdown. The biological process of early implant loss occurs around the implant in the bone and therefore needs a model that concentrates on 2 critical parameters: (1) the pre-contamination of the implant bed before implantation and (2) an intrinsic reproducibility that also results a high prevalence (> 25%) of early implant loss. No model currently exists that incorporates these two factors.

Study of periodontal and peri-implant reactions presents as complex process involving various tissues and can only be done in animal models prior to human clinical application. The type and size of the animal seem crucial [15], because no animal model represents a perfect copy of human bone and clinical conditions. A review published in 2007 clearly shows the need for experimentation on animal models compared to the various in vitro tests. Dogs, sheep, and goats seem to be the best animal models for research on the interface reaction between bone and implants [16].

The objective of this project was to develop and validate a new, reproducible preclinical model to study early implant loss, where local infection conditions would impair implant osseointegration resulting in implant failure within the first 2 months of implant placement.

## Material and methods

### Animals and ethical statement

Eight healthy adult 3-year-old female Beagle dogs (Isoquimen, Barcelona, Spain) were used in this experimental model, after the approval of the ethical statement (03/16/LU-001). The dogs’ housing, daily monitoring, and experimental procedures were conducted in the Animal Experimentation Service Facility of the Rof Codina Foundation (Lugo, Spain) by veterinarians and dental clinicians trained and accredited in laboratory animal science. During the experiment, the dogs were maintained in groups in a kennel with an indoor (15 m2) and outdoor (20 m2) area, with natural light, air renewing, and controlled temperature to 18 ± 2 °C within the indoor area. The animals were fed using a granulated dog diet twice a day using individual bowls and a free supply of water. All the experiments were performed in accordance with the Spanish and European Union regulations regarding care and use of research animals. The ARRIVE recommendations [17] were taken into account during the preparation of the manuscript.

### Study design and randomization

This study was designed as a preclinical randomized controlled trial for the comparison of two groups of treatment. The study was performed in three surgical phases using a parallel experimental design instead of a split mouth to discard any systemic effect at animal level that could jeopardize the cleaned site in an animal with an infected hemimandible. Due to the exploratory nature of this study, no sample size calculation was performed [18, 19].

### Surgical procedures

All surgical procedures were done under sterile conditions, in an animal operating theater and under general anesthesia induced by propofol (3–5 mg/kg/i.v., Propovet, Abbott), and maintained on a concentration of 2.5–4% of isoflurane (Isoba-vet®, Schering-Plough). The animals were first premedicated with medetomidine (20 mg/kg/i.m., Domtor, Esteve) and pain controlled with the administration of morphine (0.4 mg/kg/i.m., Morfina Braun 2%, B. Braun Medical). During anesthesia, the animals were continuously monitored by a veterinarian category B or C, controlling electrocardiography, capnography, pulsioxymetry, and non-invasive blood pressure. At the end of the procedures, atipamezol (50 mg/kg/i.m., Esteve) was administered to revert the effect of the medetomidine. Postoperative pain was controlled by administration of morphine (0.2 mg/kg/i.m./6 h) and meloxicam as antiinflammatory and analgesic treatment (0.2 mg/kg/i.m./SID, Metacam, Boehringer Ingelheim) during 5 days.

#### Phase 1: tooth extraction

In the first surgery, the extraction of the mandibular 2nd, 3rd, and 4th premolar (PM2-PM4) was done. The teeth were hemisected and carefully removed with elevators and forceps in a flapless approach. Prophylactic administration of cephazolin (20 mg/kg/i.v., Kurgan, Normon) and cefovezin (8 mg/kg/s.i.d/s.c., Convenia, Zoetis) was done intraoperatively.

#### Phase 2: implant placement and ligature placement

In the second surgery, 3 months after tooth extraction, a full thickness mucoperiosteal was elevated bilaterally in the mandible premolar region and a total of 48 machined implants (2.9 × 10 mm) were installed (Bone Level, Straumann AG). Three implants were placed in each side of the mandible and healing abutments of 5.5 mm in height were tightened to each implant. Before flap suturing and according to a randomization scheme, 4–0 silk ligatures were placed around the implant abutment connection in four animals (test group) as described previously by Lindhe et al. [20]. In the other 4 animals (control group), no ligature was placed, and flaps were repositioned in both groups in a non-submerged healing and sutured with absorbable suture (Vicryl, Ethicon).

#### Phase 3: implant replacement

Four weeks after implant installation and ligature placement, all implants were removed with an implant removal kit. Immediately after, new implants (3.3 × 8 mm), slightly wider, were installed with a flapless approach (Bone Level SLActive, Straumann AG) into the explanted sites without drilling and/or irrigation. Test implants were installed in theoretically infected sites and control in “non-infected sites.” Pain control was controlled routinely, but neither antibiotics were administered once ended the surgery nor plaque control in both groups.

#### Phase 4: monitoring the implants

Animals were monitored daily to check the health status and weekly to detect the failure and loss until the termination of the study.

#### Phase 5: euthanasia and histological processing

After 8 weeks of healing, the dogs were euthanized with an overdose of intravenous injection of sodium pentobarbital (40–60 mg/kg/i.v., Dolethal, Vetoquinol) previously sedated with medetomidine (30 µg/kg/i.m., Esteve). Subsequently, the lower jaws were dissected and fixed in buffered 10% formaldehyde solution at a temperature of 4 °C for 2 weeks. Blocks containing the hard and soft tissues surrounding the implant were processed following the method described by Donath and Breuner [21]. Central sections of implants and failure regions were obtained with a thickness of approximately 50 microns and stained according to the Levai Laczkó method [22]. A histologic description of the hard tissues of the failure implants sections was performed.

### Histometric analysis

Images were analyzed and captured using a motorized light microscope and a digital camera connected to a PC-based image capture system (BX51, DP71, Olympus). Implant shoulder and the first bone-implant contact (fBIC) were identified from the images and measured using a digitizing pad and an image analysis system (CellSens, Olympus). In addition, the BIC along the implant surfaces was calculated separately for buccal and lingual sides. For that purpose, a region of interest (ROI) was defined with a length of 4 mm in the center on the buccal and lingual side of each implant following a method previously published [23] and calculated as a percentage.

### Statistical analysis

Implant loss (primary outcome variable) was defined as presence or not at the implant site. The data rows were examined with the Shapiro–Wilk’s test for normal distribution. For the comparison between groups, Mann–Whitney’s *U* test was used. The α‐error was set at 0.05.

Mean values and standard deviation of secondary outcomes (fBIC and BIC) were calculated for each implant. *T*-test was used to analyze differences among variables (Sigma Plot 12.5, Systat software Inc.). *P* values < 0.05 were considered to be significant.

## Results

### Histological findings

#### Failed sites

**Fig. 1:**
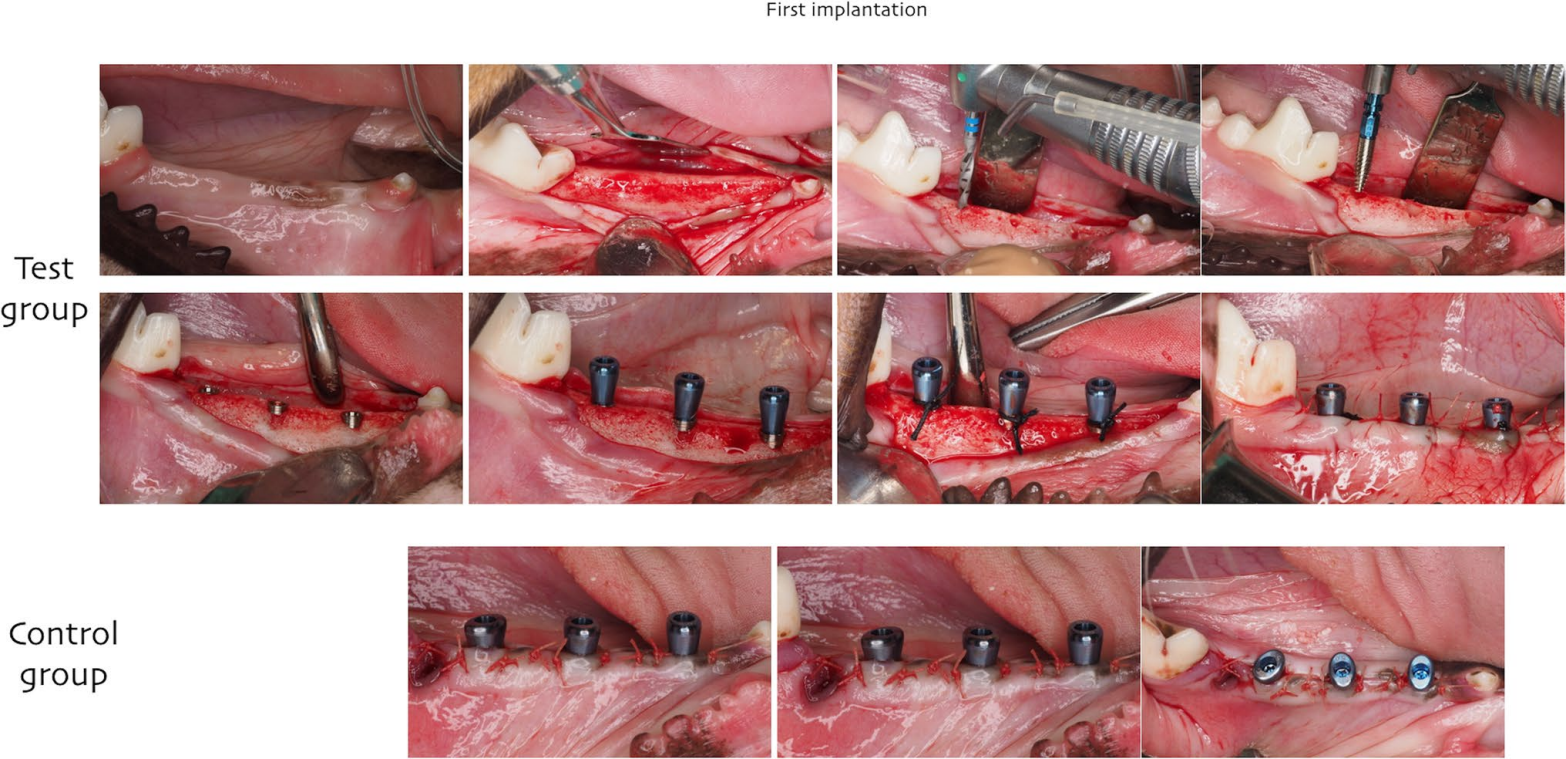
Clinical pictures showing first implant surgery and study group creation. Placing 2.9-mm diameter smooth bone level tapered implant. Ligature placed at the implant-abutment connection at the test group

**Fig. 2:**
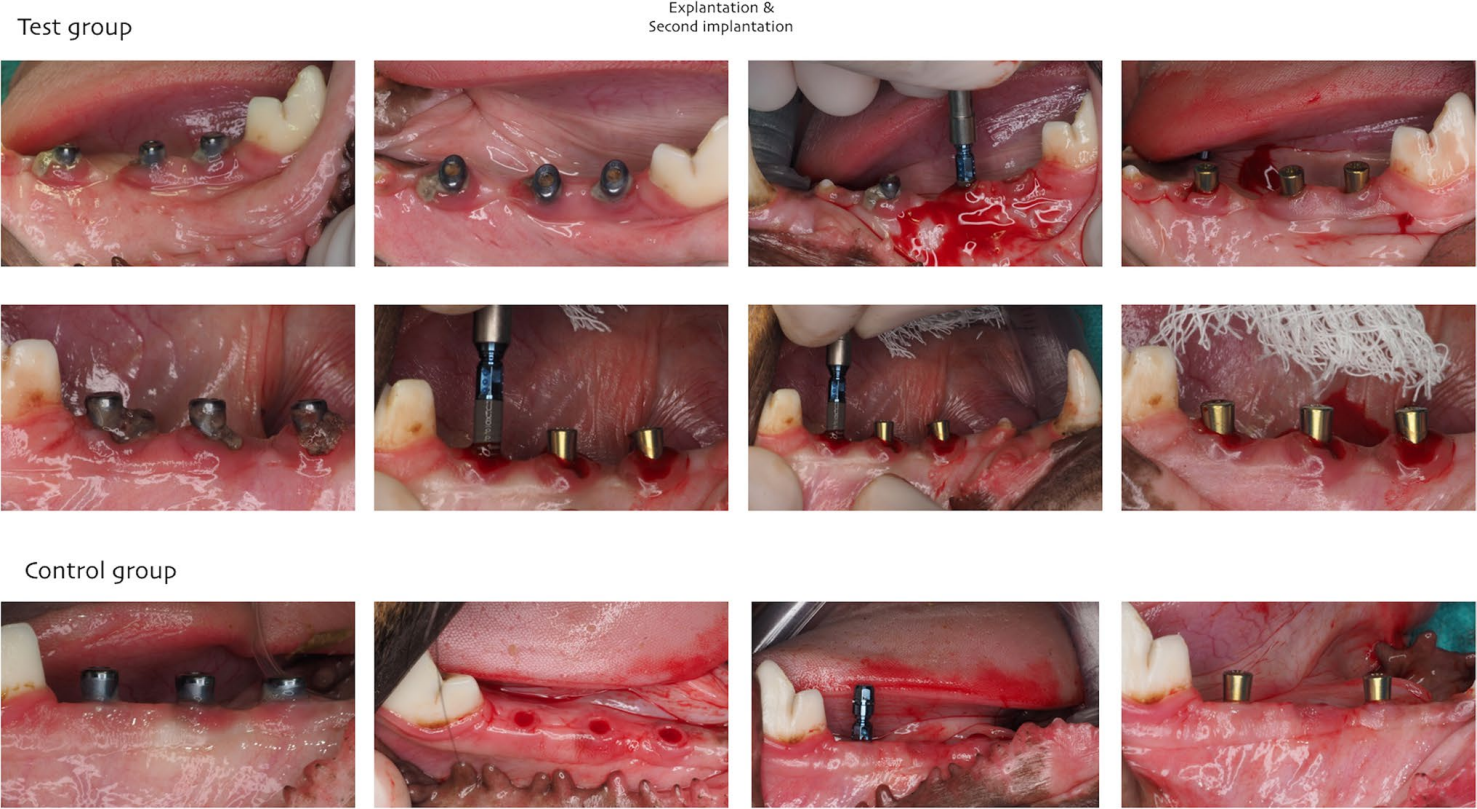
Four weeks after first implantation, explantation and new implant installation were performed. New 3.3-mm diameter bone level tapered SLActive implant. Note the amount of inflammation at the test in comparison to the control sites

**Fig. 3:**
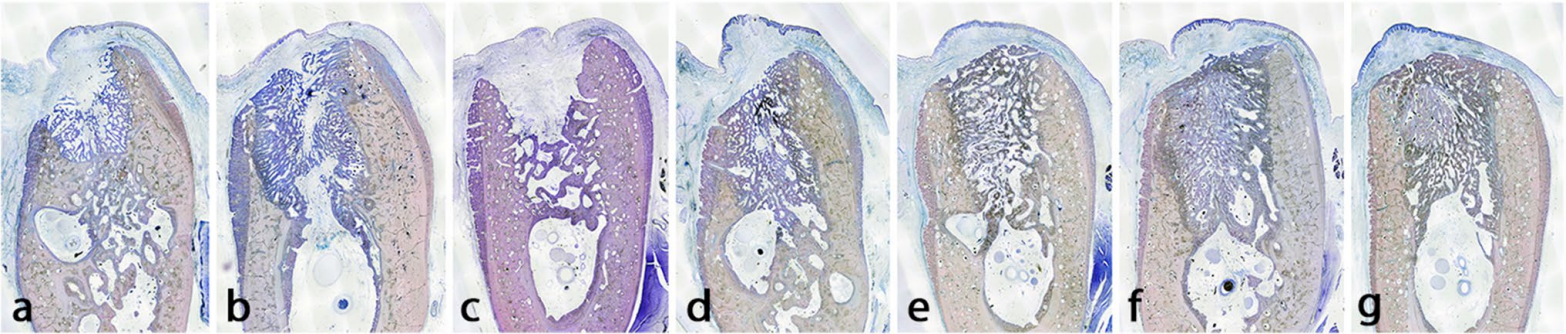
Sequence of the bone healing process in defects created around lost implants. **a** Lost in the last 3 weeks, **b** 3–4 weeks, **c**, **d** 4–5 weeks, **e** 5–6 weeks, **f** 6–7 weeks, **g** 7–8 weeks

Eight weeks after last implant installation, 13 implants were lost and 1 presented with mobility at the test group. Thus, early implant loss was recorded in 14 out of 24 implants (58.3%). In the control group, 4 out of 23 implants (17.4%). All the implants lost in the control group were in the same animal; thus, no implant loss was recorded in the other 3 control animals (Figs. 1, 2, and 3).

Screening for implant loss was performed on a weekly interval. Most of implant loss was detected within the first 5 weeks, 3 in the control (80%) and 13 (86.7%) in the test groups (Tables 1 and 2).

**Table 1:** Early implant loss at each week interval and animal studied. R and L indicate the side and position of the implant (*R* right, *L* left, 1 to 3 from mesial to distal)

Early implant loss per week								
	Week 1	Week 2	Week 3	Week 4	Week 5	Week 6	Week 7	Week 8
Control *n* = 23	0	1	0	1	1	0	1	0
Test *n* = 24	2	4	3	2	2	0	0	1(mobility)
Control 1								
Control 2	R2							
Control 3					L2			
Control 4		L3		R2			R1	
Test 1	L1			R1				
Test 2		R1	R2		R3			
Test 3		R2, L1, L2	R3		L3			
Test 4	R3		L2	L1

**Table 2:** Cumulative early implant loss at each week and final

Cumulative early implant loss									
	Week 1	Week 2	Week 3	Week 4	Week 5	Week 6	Week 7	Week 8	Total (%)
Control *n* = 23	0	1	1	2	3	3	4	4	4 (17.39%)
Test *n* = 24	2	6	9	11	13	13	13	14	14 (58.33%)

Only one implant of each group was lost in the last 2 weeks of healing. These defects presented fragments of bone being phagocyted by osteoclast-like cells, surrounded by woven bone, more differentiated in the apical portion of the defect. The coronal area showed abundant blood vessels surrounded by collagen fibers where calcium is beginning deposited in some areas. Some detritus and necrotic rests are seen, but neither lymphocytes nor neutrophils. Gingiva covered the jaw, but connective tissue was composed by thin collagen fibers that tried to cover the implant defect (Fig. 3a).

Sections representing 4 weeks of healing presented a totally developed connective tissue over the defect rich in collagen fibers disposed in groups. The bone defect was filled in all blocks, except in one, with immature bone rich in cells. This bone was holed by multiple blood vessels and between these and the bone was evident the presence of osteoblast producing new bone. Bone crest presented different images, caused by the severity of the lesion produced by the infection. In two of the sections, the original buccal crest had been substituted by a new bone of periosteal origin, with evident signs of osteoclastic resorption. In adjacent areas to periosteal, the pristine lamellar jaw bone showed an increment in porosity, compatible with bone remodeling. Only in one case the defect was not totally closed (Fig. 3b).

At 5 weeks of healing, the presence of osteoid formation surrounding the woven bone was still present. It seems that in some cases, the buccal table was partially collapsed with a reduction of the width and signs of osteoclastic activity around the buccal crest. In one section, the coronal portion of the defect was not filled of bone, showing a high number of lymphocytes and necrosis in the center and macrophages surrounding the infectious foci (Fig. 3c and d).

At 6 weeks of healing appears lamellar bone inside the defect, surrounding blood vessels. Around these new osteons, woven bone is present in a higher percentage. The remodeling of the jaw is evident by the increment of porosity of the cortical bone. The gingiva is well formed and the support connective tissue is mature presenting thick and interconnected collagen fibers (Fig. 3e).

At 7 weeks, the remodeling of the bone crest is shown and was substituted by new bone of periosteal origin and loss of width in different grades. At the coronal level, the percentage of immature bone is higher and the periphery of the defect is being more intensely remodeled in the areas that were more injured during the infection (Fig. 3f).

Finally, week 8 group showed more than 30% of the defect replaced with lamellar bone (Fig. 3g).

#### Implant sites

**Fig. 4:**
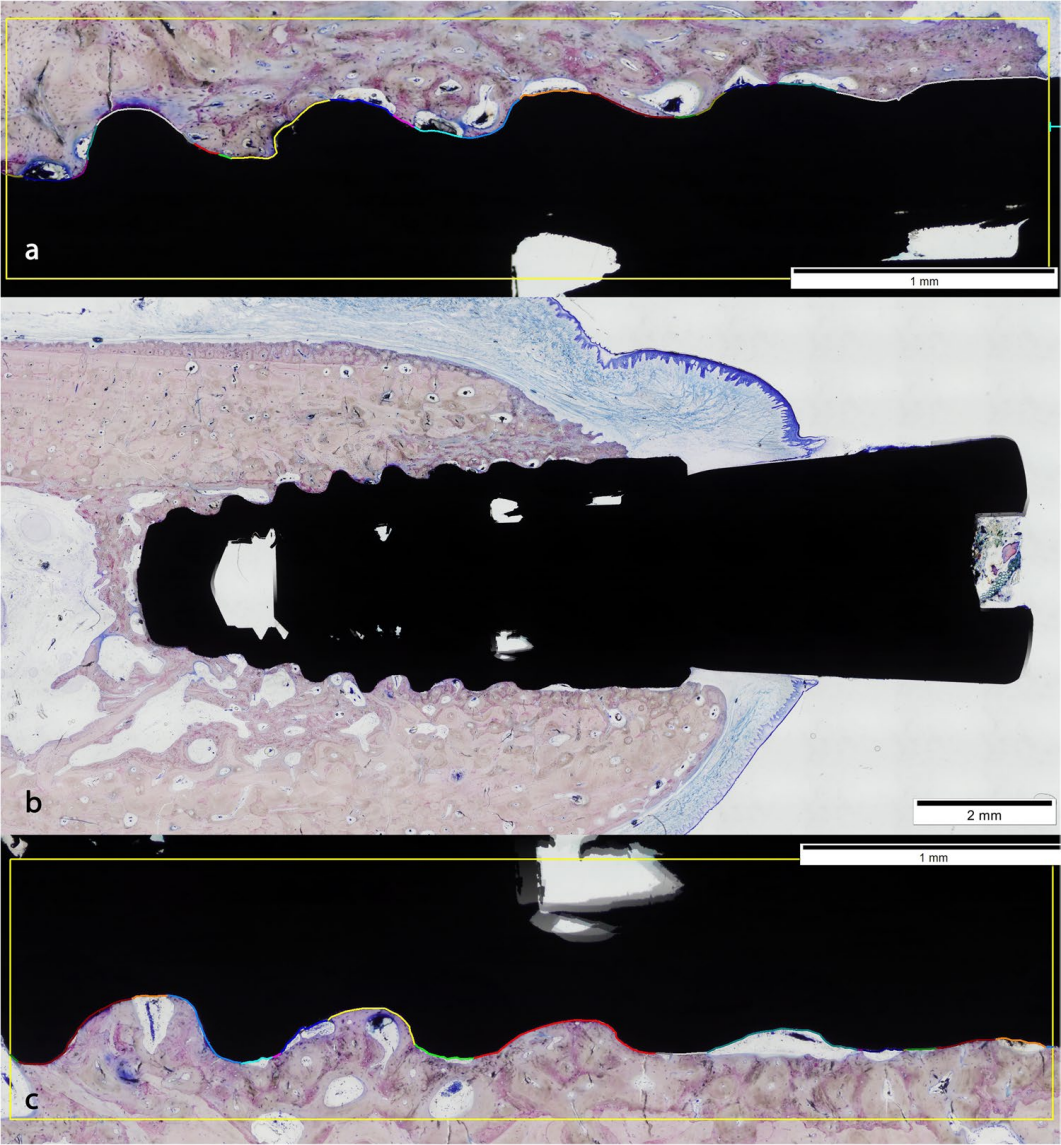
Control group (**b**). Implants were surrounded by lamellar bone in narrow contact with the bone, with abundant blood vessels. Crestal bone loss in buccal side was limited. Measurements of the bone implant contact (BIC) in the buccal (**a**) and lingual (**c**) side

**Fig. 5:**
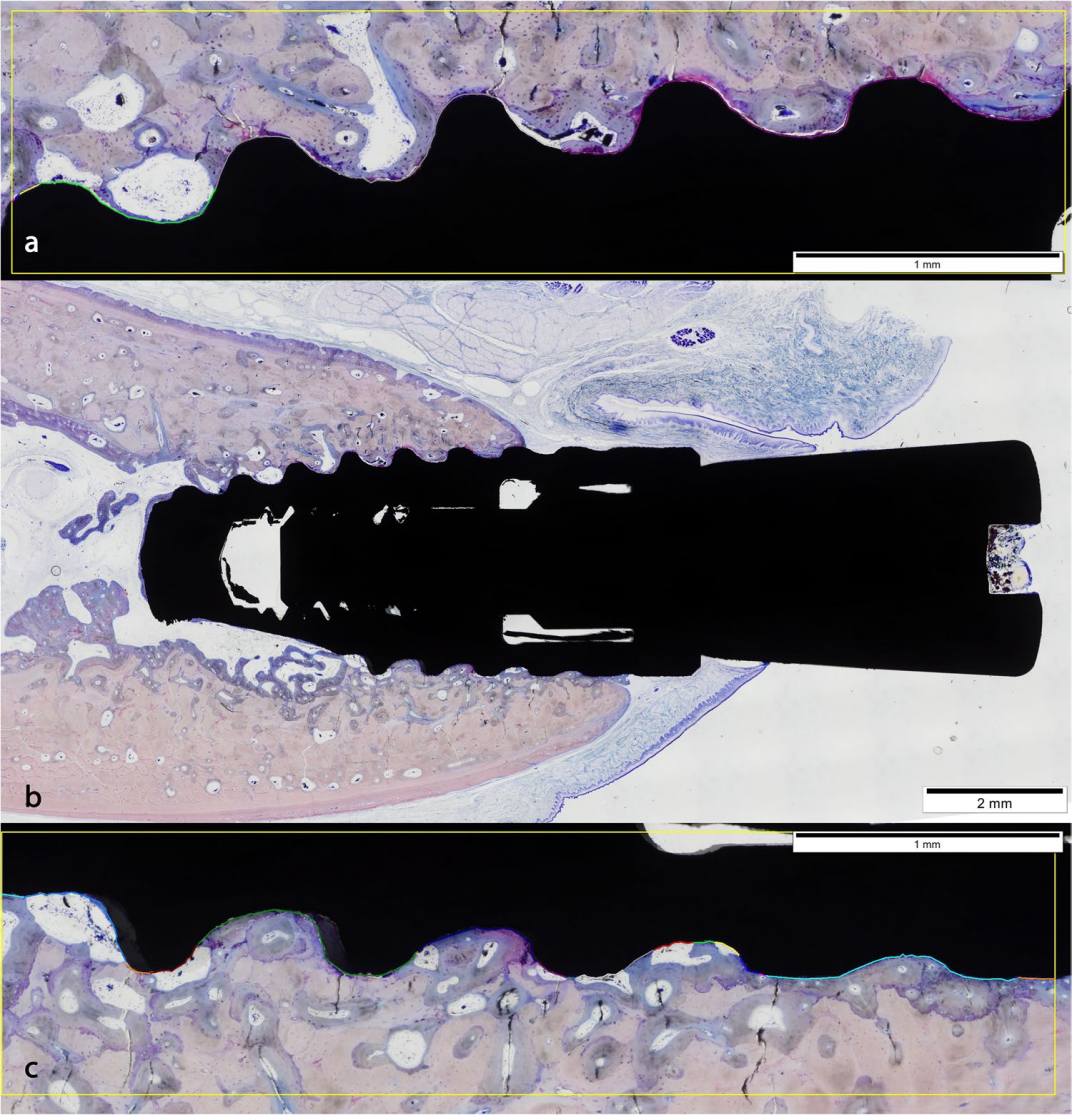
Test group (**b**). Implants showed an increment in the remodeled bone (blue intense) with a higher bone loss around buccal crest. BIC in buccal (**a**) and lingual (**c**) side

Four implants presented severe signs of infection, two of each group. The healed implants showed typical secondary osteons with concentric lamellae and a central haversian canal in the lamellar bone around them, with bone remodeling around crestal bone (Figs. 4 and 5).

### *Histometric analysis *(*Table **3*)

**Table 3:** Histometric analysis

Histometric analysis		
Control *n* = 15	S-fBIC	BIC
	1.4 ± 0.86	58.64 ± 11.59
Test *n* = 8	2.3 ± 1.42	73.25 ± 5.28
*p*	0.048	0.001

#### Distance between implant shoulder and the first bone implant contact (S-fBIC)

The distance between the implant shoulder and the buccal first bone implant contact (fBIC) was 2.3 mm (*SD* = 1.42) in the test group and 1.4 mm (*SD* = 0.86) in the control group. Difference in the mean value of the groups was statistically significant (*P* = 0.048).

#### Bone implant contact (BIC)

The bone implant contact (BIC) was 73.25% (*SD* = 5.28) in the test group and 58.64% (*SD* = 11.59) in the control group. Differences in the median values between groups were statistically significant (*P* = 0.001).

## Discussion

The present preclinical study was designed to develop and validate a reliable preclinical model to study early implant loss. It was shown that implants placed into infected implant sites have higher risk of no osseointegration and implant loss.

Derks et al. (2015) showed that the risk of placing implants in patients with active periodontal disease increases 4 times the risk of early implant loss [7]. This is in agreement to the obtained results of the present investigation, since the test group showed an increased risk of early implant loss. In the present study, a high percentage or early implant loss was detected in the control group, 17.4% of implants. This is high number if it is compared to the literature. The reason for high implant loss in the control group may be due to several aspects.

Some publications that have shown the survival of implants placed on failed implant sites have more early loss. A recent retrospective clinical study analyzed the survival of dental implants placed in sites of previously failed implants [24]. One hundred and seventy five of 10,096 implants in 98 patients were replaced by another implant at the same location (159, 14, and 2 implants at second, third, and fourth surgeries, respectively). A significantly greater percentage of lost implants was placed in sites with low bone quantity. There was a significant difference (*P* = 0.032) in the survival rates between implants that were inserted for the first time (94%) and implants that replaced those that had been lost (73%). This corresponds to a 27% early implant loss of implants placed in re-operated zones.

Grossmann and Levin [25] observed that 9 of 31 (29%) re-operated implants failed in their study. Machtei et al. [26] reported an overall survival rate of 83.5% for reoperated dental implants. Kim et al. found a lower failure rate (11.7%) for reimplantation [8], similar to the results of Alsaadi et al. [27] with 12.1% failure rates (7 of 58 implants), and Wagenberg and Froum [9] only 3.92% (2 of 51).

Thus, the data reported in the abovementioned studies are in accordance to the results obtained in the present investigation in the control group. It is important also to point out that the present investigation analyzed early implant loss in an immediate re-implantation model, and the mentioned studies were in a delayed protocol.

Although ligature-induced contamination was not applied in the control sites, those areas could be “partially contaminated” since minor inflammation and plaque were observed at the second implantation in some locations. Thus, minor infection-inflammation may induce also higher early implant loss as compared to the standards reported in the literature. However, there is no evidence of inflammatory cells in the soft tissues that surround the control implants that arrived to termination.

Differences are notated between an acute induced infection like in this study and a peri-implant infection. Experimental infection is based on a reaction to a foreign body and subsequent accumulation of bacterial plaque on the implants, accelerating the natural process that occurs clinically [28, 29].

Another explanation of high early implant loss on control sites could be related to the timing of the second implant placement. There is no data in the literature regarding implant survival rates of immediate implants placed in explanted sites. Moreover, in the present study, implants were placed in an area just 4 weeks after first implant surgery. After 4 weeks, some partial bone necrosis due to bone remodeling may be present and potentially jeopardizing the early healing of the new implants [30, 31].

Finally, some authors stated evidence of cluster patterns (more than one implant failure per patient) among patients with implant failure [32–34]. In our study, all the control implants were lost in a same animal.

However, both implants placed in the phase 3 had the same characteristics of hydrophilicity. That is because we do not consider that the type of surface may have had influence in the results.

One interesting fact was that the test implants showed a significant higher bone implant contact percentage and coronal bone loss compared with the control group. The increment in the distance between the implant shoulder and the first implant contact looks reasonable if it is considered that the microbiota present in the soft tissue that surrounds the implant may induce a bone resorption. However, an explanation to the increment in the bone implant contact must be found.

Levin et al. in 2011 induced infection around dental implants in 2 dogs using silk ligatures. Then, those implants were explanted and placed in pristine bone. In infected sockets placed new implants, not obtaining differences in the BIC values between groups. Authors concluded with limitations that osseointegration could be achieved around infected sites and contaminated implants [35]. Similar results were obtained by Kim et al. in a similar model inducing the infection leaving exposed the 4-mm upper part of implants for later placing the infected implants in a corrected position in the same or in pristine bone [36]. This study was based in a previous study of Kolonidis et al. who found osseointegration of infected implants placed in pristine bone [37].

Isidor in 1997 stated an increment in BIC if considered only the apical portions of the implant [38]. However, the method of measurement of BIC in his study was different because he considered the exposed portion of the implant in the total BIC and only the endosseal portion in apical BIC.

Delgado-Ruiz et al. [39] reviewed the occlusal forces on the peri-implant-bone interface stability. They concluded that local and individual factors can influence the strength of the osseointegration and the biological effects of the occlusal load. Romanos in 2003 showed that loading increased the ossification around endosseous implants [40]. The theory was that occlusal loading may increase the blood circulation in bone, enhancing bone metabolism and promoting the bone remodeling. Although our implants were not loaded, we observed an increment in the bone remodeling around test implants. We have two theories about it: first and more feasible, probably our test implants were more exposed to the occlusal loads because the loss of crestal bone and this could be the cause of the increment in the BIC. If we consider that no occusal forces were present, it could be hypothesized that remodeling might be induced during the active phase of progression of the perimplantitis and, once the infected implant is removed, the new interface converted in a resting non progressive lesion, as was precluded by Lindhe in 1992 [20]. How to modulate this effect could be interesting to increase the bone implant contact around implants with problems.

The aim of this study is to propose an experimental model for the investigation of early loss of dental implants. With the results of our study, it seems that the model may work for the study of early loss of implants after placement or justify in part the clinical pathogenesis of the process. The same model may be suitable and easily adaptable for the investigation of other factors in the research on oral implantology.

## References

[1] Pjetursson BE, Asgeirsson AG, Zwahlen M, Sailer I (2014). Improvements in implant dentistry over the last decade: comparison of survival and complication rates in older and newer publications. Int J Oral Maxillofac Implants.

[2] Tomasi C, Etiology Derks J (2022). Occurrence, and consequences of implant loss. Periodontol 2000..

[3] Monje A, Nart J (2022). Management and sequelae of dental implant removal. Periodontol 2000.

[4] Schwarz F, Jepsen S, Obreja K, Galarraga-Vinueza ME, Ramanauskaite A (2022). Surgical therapy of peri-implantitis.. Periodontol 2000.

[5] Esposito M, Hirsch JM, Lekholm U, Thomsen P (1998). Biological factors contributing to failures of osseointegrated oral implants II Etiopathogenesis. Eur J Oral Sci.

[6] Sakka S, Baroudi K, Nassani MZ (2012). Factors associated with early and late failure of dental implants. J Investig Clin Dent.

[7] Derks J, Håkansson J, Wennström JL, Tomasi C, Larsson M, Berglundh T (2015). Effetiveness of implant therapy analyzed in a Swedish population: early and late implant loss. J Dent Res.

[8] Kim YK, Park JY, Kim SG, Lee HJ (2010). Prognosis of the implants replaced after removal of failed dental implants. Oral Surg Oral Med Oral Pathol Oral Radiol Endod.

[9] Wagenberg B, Froum SJ (2006). A retrospective study of 1925 consecutively placed immediate implants from 1988 to 2004. Int J Oral Maxillofac Implants.

[10] Gianserra R, Cavalcanti R, Oreglia F, Manfredonia MF, Esposito M (2010). Outcome of dental implants in patients with and without a history of periodontitis: a 5-year pragmatic multicentre retrospective cohort study of 1727 patients. Eur J Oral Implantol.

[11] Pommer B, Frantal S, Willer J, Posch M, Watzek G, Tepper G (2011). Impact of dental implant length on early failure rates: a meta-analysis of observational studies. J Clin Periodontol.

[12] Kolonidis SG, Renvert S, Hämmerle CH, Lang NP, Harris D, Claffey N (2003). Osseointegration on implant surfaces previously contaminated with plaque An experimental study in the dog. Clin Oral Implants Res.

[13] Alhag M, Renvert S, Polyzois I, Claffey N (2008). Re-osseointegration on rough implant surfaces previously coated with bacterial biofilm: an experimental study in the dog. Clin Oral Implants Res.

[14] Mohamed S, Polyzois I, Renvert S, Claffey N (2010). Effect of surface contamination on osseointegration of dental implants surrounded by circumferential bone defects. Clin Oral Implants Res.

[15] Kantarci A, Hasturk H (2000). Van Dyke TE (2015) Animal models for periodontal regeneration and peri-implant responses. Periodontol.

[16] Pearce AI, Richards RG, Milz S, Schneider E, Pearce SG (2007). Animal models for implant biomaterial research in bone a review. Eur Cell Mater.

[17] Kilkenny C, Browne WJ, Cuthill IC, Emerson M, Altman DG (2010). Improving bioscience research reporting: the ARRIVE guidelines for reporting animal research. PLoS Biol.

[18] Berglundh T, Gotfredsen K, Zitzmann NU, Lang NP, Lindhe J (2007). Clin oral implants Res..

[19] Sandvik L, Erikssen J, Mowinckel P, Rødland EA. Stat Med. (1996) A method for determining the size of internal pilot studies. 1996 Jul 30;15(14):1587–90 10.1002/(SICI)1097-0258(19960730)15:14<1587::AID-SIM279>3.0.CO;2-F. 10.1002/(SICI)1097-0258(19960730)15:14<1587::AID-SIM279>3.0.CO;2-F 8855484

[20] Lindhe J, Berglundh T, Ericsson I, Liljenberg B, Marinello C (1992). Experimental breakdown of peri-implant and periodontal tissues A study in the beagle dog. Clin Oral Imp Res.

[21] Donath K, Breuner G (1982). A method for the study of undecalcified bones and teeth with attached soft tissues The Sage-Schliff (sawing and grinding) technique. J Oral Pathol.

[22] Jenö L, Géza LA (1975). A simple differential staining method for semi-thin sections of ossifying cartilage and bone tissues embedded in epoxy resin. Mikroskopie.

[23] Thoma DS, Benic GI, Muñoz F, Kohal R, Sanz Martin I, Cantalapiedra AG, Hämmerle CHF, Jung RE (2015). Histological analysis of loaded zirconia and titanium dental implants: an experimental study in the dog mandible. J Clin Periodontol.

[24] Chrcanovic BR, Kisch J, Albrektsson T, Wennerberg A (2017). Survival of dental implants placed in sites of previously failed implants. Clin Oral Implants Res.

[25] Grossmann Y, Levin L (2007). Success and survival of single dental implants placed in sites of previously failed implants. J Periodontol.

[26] Machtei EE, Mahler D, Oettinger-Barak O, Zuabi O, Horwitz J (2008). Dental implants placed in previously failed sites: survival rate and factors affecting the outcome. Clin Oral Implants Res.

[27] Alsaadi G, Quirynen M, van Steenberghe D (2006). The importance of implant surface characteristics in the replacement of failed implants. Int J Oral Maxillofac Implants.

[28] Schwarz F, Derks J, Monje A, Wang HL (2018). Peri-implantitis. J Clin Periodontol..

[29] Camps-Font O, Figueiredo R, Valmaseda-Castellón E, Gay-Escoda C (2015). Postoperative infections after dental implant placement: prevalence, clinical features, and treatment. Implant Dent..

[30] Berglundh T, Abrahamsson I, Lang NP, Lindhe J (2003). De novo alveolar bone formation adjacent to endosseous implants. Clin Oral Implants Res.

[31] Abrahamsson I, Berglundh T, Linder E, Lang NP, Lindhe J (2004). Early bone formation adjacent to rough and turned endosseous implant surfaces An experimental study in the dog. Clin Oral Implants Res.

[32] Jemt T, Häger P (2006). Early complete failures of fixed implant-supported prostheses in the edentulous maxilla: a 3-year analysis of 17 consecutive cluster failure patients. Clin Imp Dent Relat Res.

[33] Schwartz-Arad D, Laviv A, Levin L (2008). Failure causes, timing, and cluster behavior: an 8-year study of dental implants. Implant Dent.

[34] Tonetti MS (1999). Determination of the success and failure of root form osseointegrated dental implants. Adv Dent Res.

[35] Levin L, Zigdon H, Coelho PG, Suzuki M, Machtei EE (2011). Reimplantation of dental implants following ligature-induced peri-implantitis: a pilot study in dogs. Clin Implant Dent Relat Res.

[36] Kim YT, Cha JK, Park JC, Jung UW, Kim CS, Cho KS, Choi SH (2012). In situ dental implant installation after decontamination in a previously peri-implant diseased site: a pilot study. J Periodontal Implant Sci.

[37] Kolonidis SG, Renvert S, Hämmerle CHF, Lang NP, Harris D, Claffey N (2003). Osseointegration on implant surfaces previously contaminated with plaque. An experimental study in the dog. Clin Oral Impl Res.

[38] Isidor F (1997). Histological evaluation of peri-implant bone at implants subjected to occlusal overload or plaque accumulation. Clin Oral Impl Res..

[39] Delgado-Ruiz RA, Calvo-Guirado JL, Romanos GE (2000). (2019) Effects of occlusal forces on the peri-implant-bone interface stability. Periodontol.

[40] Romanos GE, Toh CG, Siar CH, Wicht H, Yacoob H, Nentwig G (2003). Bone-implant interface around titanium implants under different loading conditions a histomorphometrical analysis in the Macaca fascicularis monkey. J Periodontol..

